# Two Paralogous Genes Encoding Auxin Efflux Carrier Differentially Expressed in Bitter Gourd (*Momordica charantia*)

**DOI:** 10.3390/ijms18112343

**Published:** 2017-11-06

**Authors:** Yi-Li Li, Yun-Shan Lin, Pung-Ling Huang, Yi-Yin Do

**Affiliations:** 1 Department of Horticulture & Landscape Architecture, National Taiwan University, Taipei 10617, Taiwan; yili2emily@gmail.com (Y.-L.L.); linashango@gmail.com (Y.-S.L.); pungling@ntu.edu.tw (P.-L.H.); 2 Graduate Institute of Biotechnology, Chinese Culture University, Taipei 11114, Taiwan

**Keywords:** auxin transporter, sequence identity, phylogenetic analysis, gene expression, fruit development, auxin induction

## Abstract

The phytohormone auxin regulates various developmental programs in plants, including cell growth, cell division and cell differentiation. The auxin efflux carriers are essential for the auxin transport. To show an involvement of auxin transporters in the coordination of fruit development in bitter gourd, a juicy fruit, we isolated novel cDNAs (referred as McPIN) encoding putative auxin efflux carriers, including *McPIN1*, *McPIN2* (allele of *McPIN1*) and *McPIN3*, from developing fruits of bitter gourd. Both *McPIN1* and *McPIN3* genes possess six exons and five introns. Hydropathy analysis revealed that both polypeptides have two hydrophobic regions with five transmembrane segments and a predominantly hydrophilic core. Phylogenetic analyses revealed that McPIN1 shared the highest homology to the group of *Arabidopsis*, cucumber and tomato PIN1, while McPIN3 belonged to another group, including *Arabidopsis* and tomato PIN3 as well as PIN4. This suggests different roles for McPIN1 and McPIN3 in auxin transport involved in the fruit development of bitter gourd. Maximum mRNA levels for both genes were detected in staminate and pistillate flowers. *McPIN1* is expressed in a particular period of early fruit development but *McPIN3* continues to be expressed until the last stage of fruit ripening. Moreover, these two genes are auxin-inducible and qualified as early auxin-response genes. Their expression patterns suggest that these two auxin transporter genes play a pivotal role in fruit setting and development.

## 1. Introduction

Auxins are plant hormones that mediate many aspects of plant growth and development. Auxins stimulate cell enlargement, stem growth, cell division in the cambium, differentiation of phloem and xylem, root initiation and growth of flower parts. Furthermore, these hormones delay leaf senescence and are involved in apical dominance and leaf abscission. Auxin can induce fruit setting and growth in some fruits, assimilate partitioning and promote flowering in Bromeliads and female traits in dioecious flowers [1]. As early as 1926, Cholodny and Went proposed that the gravitropic curvature of plant organs was dependent upon lateral transport of the plant hormone indole-3-acetic acid (IAA)—auxin—from the upper to the lower side of a responding organ, resulting in different auxin concentration on the two sides of the organ. Different auxin levels result in different elongation rates and, thus, change the growth curvature [2].

As auxin is synthesized in young leaves of the shoot system and transported downward to the root tip, auxin moves in a basal-to-apical (acropetal) direction in the root [3]. The strongly polar process of auxin movement through the components of cells is thought to involve both passive diffusion and active transport. Therefore, the existence of a set of asymmetrically distributed molecules is required for the influx and efflux of auxin [1]. In this directional transport, auxin is mobilized by adenosine triphosphate-driven transporter (ATP Binding Cassette subfamily B; ABCBs), gradient-driven carriers (PIN-FORMED; PINs) and amino acid/auxin permease family of proton-driven symporters (AUXIN RESISTANT1/LIKE AUX1, AUX1/LAXes). There are at least 15 different carriers belonging to these three classes of auxin transporters in *Arabidopsis* [4,5].

In *Arabidopsis*, the PIN family consists of eight members and is divided into two subclades: full-length PINs (AtPIN1, 2, 3, 4 and 7) with polar localization on plasma membrane (PM) and short PINs (AtPIN5, 6 and 8) localized to endomembranes with a shorter central hydrophilic loop [6]. AtPIN1 acts as the auxin efflux carrier in *Arabidopsis* vascular tissue [7]. Re-localization of the AtPIN1 plays a role in phototropic responses [8]. Direct involvement of AtPIN1 in organogenesis and embryogenesis has also been demonstrated [9]. AtPIN2/EIR1/AGR1 mediates auxin reflux flows from the root apex [10]. AtPIN3 functions in the lateral redistribution of auxin and is present in the stem endodermal layer and in columella cells of the root cap [11]. Moreover, AtPIN3 functions during gravity response in roots and in hypocotyl by polarization [12]. Mobilization of auxin out of the root meristem is dependent on AtPIN3 and AtPIN4 [13]. AtPIN7 plays a role in forming and maintaining apical−basal auxin gradients that are essential for establishing embryonic polarity and functions in root acropetal auxin transport. This first presents in columella cells, and relocates during gravitropic stimulation [13,14]. AtPIN5 and AtPIN8 are localized to the endoplasmic reticulum (ER) and mediate auxin flow from the cytosol to the lumen of the ER to regulate subcellular homeostasis of auxin [6,15]. AtPIN6 at the PM and ER mediates both transportation of auxin across the PM and intracellular homeostasis of auxin during primary, lateral and adventitious root organogenesis and other developmental processes, such as shoot apical dominance [16,17].

Very little information is known about the molecular basis of auxin transport during fruit set and development until recently. Ten tomato *PIN* (*SlPIN1* to *10*) genes were identified and their expression patterns are heterogeneous [18]. The expression of tomato auxin efflux carrier genes *SlPIN1* (referred as *SlPIN9* in [19]) and *SlPIN2* (referred as *SlPIN4* in [19]) was highest in the very young fruit 2 and 4 days after anthesis, respectively. This suggests that these two proteins play important roles in the fruit setting. Furthermore, the expression of the *SlPIN2* promoter in transgenic tomatoes was restricted to ovules and young seeds 0 and 4 days after anthesis, which suggests that SlPIN2 play roles in exporting auxin synthesized in young seeds. *SlPIN1*, which had relatively high expression in the whole early tomato fruit and declined to lower levels by 15 days after anthesis, might be responsible for the subsequent transport of auxin into peduncles [20]. Eight *SlPIN* genes were expressed in fruit and could be divided into three classes: *SlPIN5* in seeds/locular tissue; *SlPIN1*, *SlPIN4*, *SlPIN7* and *SlPIN8* in placenta; and *SlPIN6* in pericarp [18]. Specific silencing of *SlPIN4*, which is much more highly expressed in ovary and fruit than any other *SlPIN* genes, leads to parthenocarpic fruits. This result indicates that SlPIN4 plays a major role in the auxin regulation of tomato fruit set [19].

To study the relationship between auxin polar transport and fruit development in Cucurbitaceae, we isolated cDNAs encoding the auxin efflux carrier homologue using *AtPIN1* and *AtPIN2* gene fragments as probes for screening. Here, we report a primary feature of putative auxin efflux carrier genes from bitter gourd and their gene expression patterns during bitter gourd fruit development.

## 2. Results

### 2.1. Isolation and Characterization of Auxin Efflux Carrier cDNAs from Bitter Gourd

**Figure 1:**
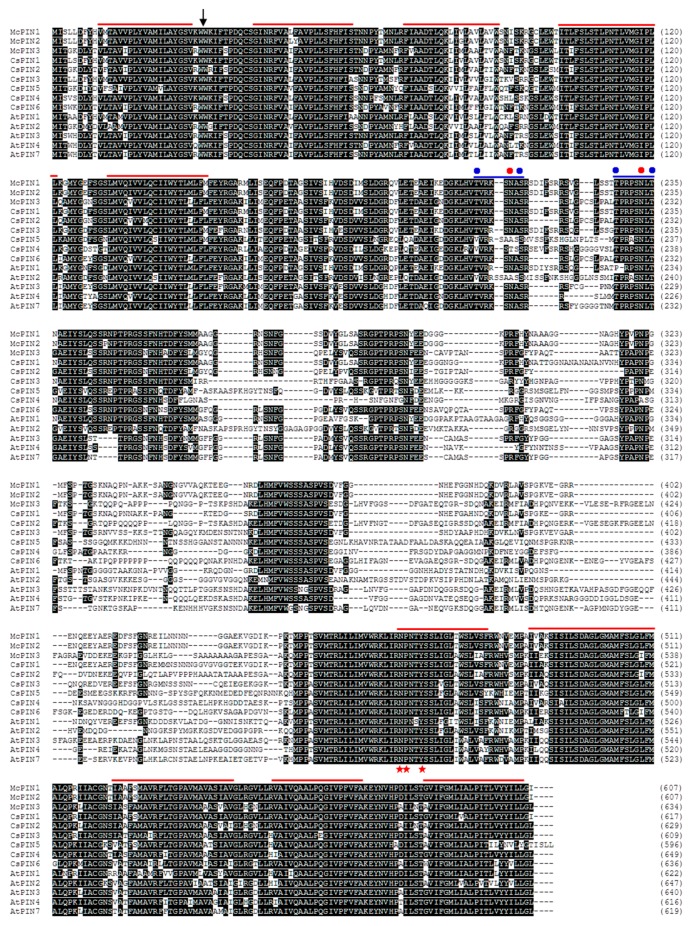
Comparison of amino acid sequences among auxin efflux carriers from bitter gourd (McPIN1 to 3), and cucumber (CsPIN1 to 6). Gaps have been introduced to maximize the alignments. Amino acid sequences were aligned using the AlignX program of Vector NTI Suite 10.0 software. An arrow indicates the cleavage site of a potential signal peptide analyzed by SignalP 3.0 [21]. Conserved transmembrane domains are indicated by red bars above sequences. Putative clusters (blue bar) of glycosylation (red dot) and two phosphorylation sites (blue dot) are indicated. Red stars indicate the internalization motif NPXXY [22]. GenBank accession numbers are AF247004 (McPIN1), AF246995 (McPIN2), AF247005 (McPIN3), AB085897 (CsPIN1), and AF539049–AF539053 (CsPIN2 to 6).

**Figure 2:**
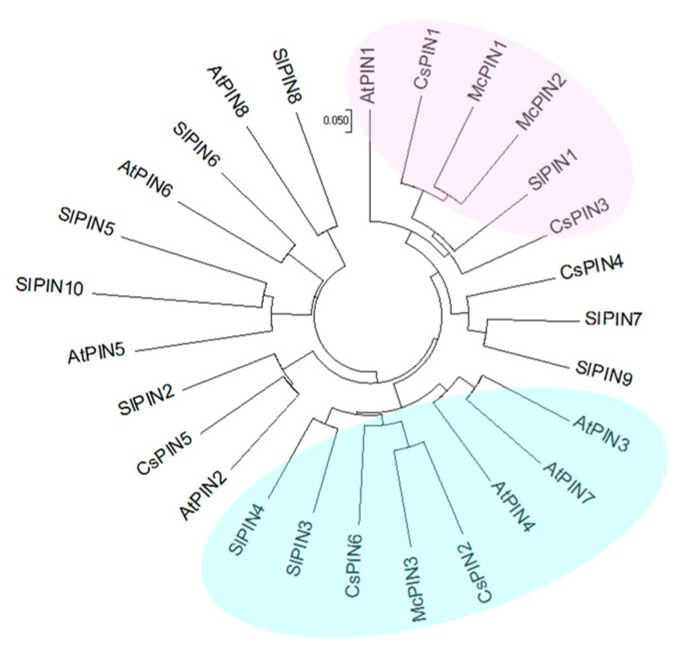
Amino acid sequence-based dendrogram among auxin efflux carriers from bitter gourd (McPIN1 to 3), cucumber (CsPIN1 to 6), and tomato (SlPIN1 to 10). GenBank accession numbers are AF247004 (McPIN1), AF246995 (McPIN2), AF247005 (McPIN3), AB085897 (CsPIN1), AF539049–AF539053 (CsPIN2 to 6), and HQ127074–HQ127083 (SlPIN1 to 10). Amino acid sequences were aligned using the AlignX program of Vector NTI Suite 10.0 software. Phylogenetic analysis was performed with the Neighbor-Joining program in Vector NTI Suite 9.0 software and output as circle by MEGA 7.0 package ([23], http://www.megasoftware.net). Scale bar indicates the number of amino acid substitutions per site.

To isolate the fruit-related auxin efflux carrier genes, the cDNA library constructed by poly(A)^+^ RNA extracted from fruit of bitter gourd was screened using *Arabidopsis AtPIN1* and *AtPIN2* fragments as probes. Among the cDNA clones obtained, pMAEC28, pMAEC43 and pMAEC93 were selected for complete nucleotide sequence analysis. Properties of these three cDNAs and deduced polypeptides are shown in Table 1. The cDNA in pMAEC28 is probably derived from an alternative splicing product, which possesses a 432 bp intron (Figure S1). The length of the 5′-untranslated region (5′-UTR) in pMAEC43 cDNA is 222 bp shorter than that in pMAEC28. After neglecting the intron and the longer 5′-UTR, pMAEC28 cDNA shares 99.7% identity of the nucleotide sequence with pMAEC43 cDNA. Therefore, pMAEC43 cDNA was used as the probe for Southern and Northern blot analysis. The encoded polypeptide by pMAEC93 cDNA possesses 27 more residues than pMAEC43 cDNA does. Hydropathy analysis revealed that both these polypeptides have two hydrophobic regions with five N-terminal (residues 1–151) and five C-terminal (residues 461–605) transmembrane segments as well as a predominantly hydrophilic core extending from residues 152–460 (Figure 1). Furthermore, amino acid sequence analysis using the program SignalP (V3.0) indicates the possibility of a signal peptide spanning from residues 1–30. The cleavage site of a potential amino-terminal signal peptide is between Trp^30^ and Trp^31^. The amino acid sequence homology among McPIN1 (corresponding to pMAEC43 cDNA), McPIN2 (corresponding to pMAEC28 cDNA), McPIN3 (corresponding to pMAEC93 cDNA) and PINs from *Arabidopsis*, excluding the short PINs of PIN5 and PIN8, is in the range of 43.9–84.2% (Table 2). McPIN1 and McPIN2 share about 73% amino acid sequence homology with AtPIN1. Moreover, as indicated in the dendrogram, the amino acid sequences of McPIN1 and McPIN2 share the highest homology with those of CsPIN1 and SlPIN1, indicating that *McPIN1* and *McPIN2* are orthologs of auxin efflux carrier *PIN1* in *Arabidopsis*. On the other hand, McPIN3 shares approximately 72% sequence homology with AtPIN3, AtPIN4 and AtPIN7 (Table 2), resulting in it being classified into the group composed of SlPIN3, SlPIN4, CsPIN2, CsPIN6 and AtPIN3/4/7 (Figure 2).

### 2.2. Genome Organization of Auxin Efflux Carrier Genes in Bitter Gourd

**Figure 3:**
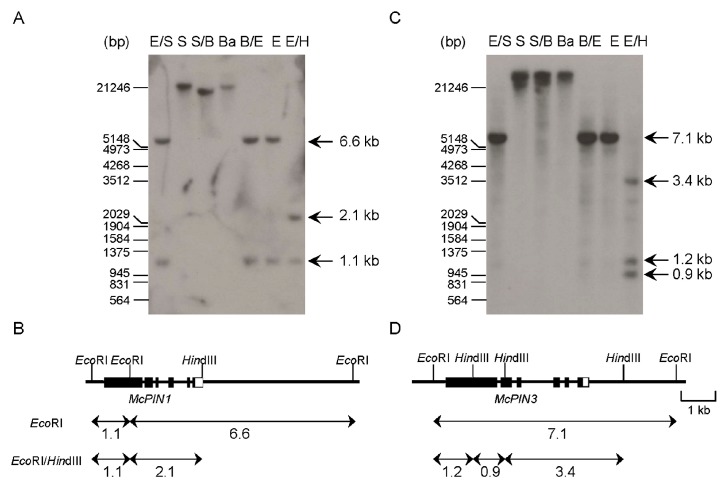
Southern blot analysis using auxin efflux carrier cDNAs from bitter gourd as probe. Genomic DNA of bitter gourd was hybridized with ^32^P-labeled insert of cDNA clone pMAEC43 for *McPIN1* (**A**,**B**) and pMAEC93 for *McPIN3* (**C**,**D**). Each lane contained 20 µg genomic DNA digested with *Eco*RI and *Sal*I (E/S), *Sal*I (S), *Sal*I and *Bam*HI (S/B), *Bam*HI (Ba), *Bam*HI and *Eco*RI (B/E), *Eco*RI (E), *Eco*RI and *Hin*dIII (E/H) and subjected to gel blot hybridization. Membrane was hybridized at 65 °C and washed at high stringency. Diagrams of the restriction maps and hybridization signals for *McPIN1* (**B**) and *McPIN3* (**D**) are presented according to the restriction patterns of cDNAs and Southern blot analyses. The locations of both cDNAs are shown by black rectangular boxes. Hybridized signals are indicated by arrows with double heads and numbers beneath arrows are sizes of the fragments.

Both cDNAs in pMAEC43 and pMAEC93 were used as gene-specific probes to perform Southern blot analyses. According to the results of Southern blot analyses, there was no *Bam*HI and *Sal*I cutting sites in the gene body of *McPIN1* and *McPIN3*. These two genes are located in different fragments after being digested with *Sal*I, *Sal*I and *Bam*HI, and *Bam*HI (Figure 3A,C). Alignment of cDNA and genomic sequences of *McPIN1* and *McPIN3* indicated that both genes contain six exons and five introns (Figure 3B,D). The lack of polymorphic fragments in Southern blot analysis indicated that both *McPIN1* and *McPIN3* genes are present in one copy per haploid genome of bitter gourd.

### 2.3. Gene Expression of Auxin Efflux Carrier during Fruit Ripening in Bitter Gourd

**Figure 4:**
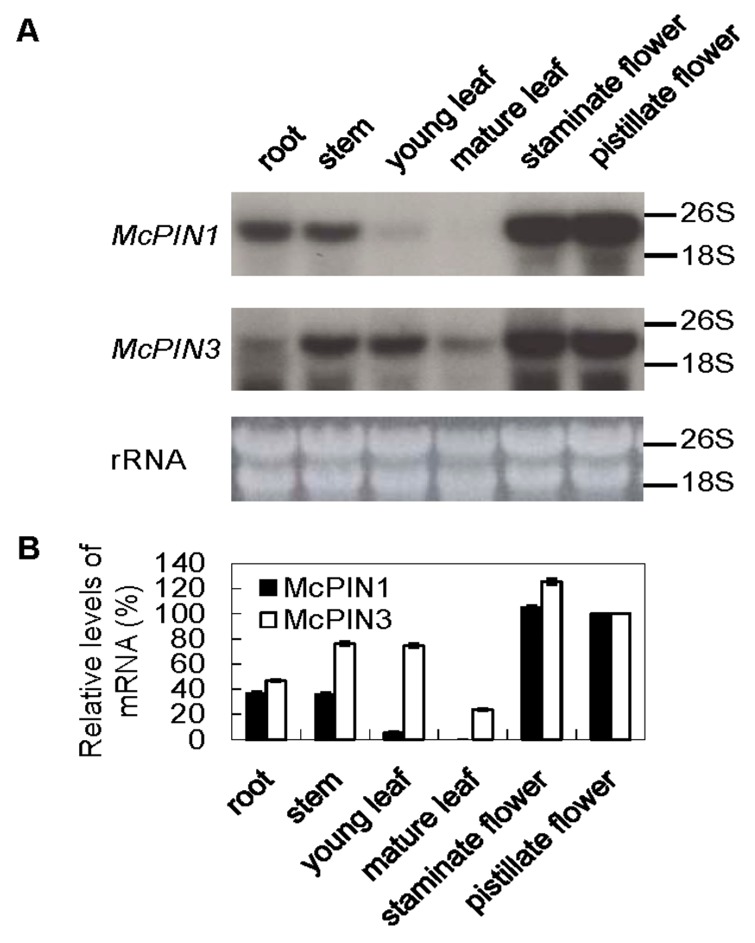
Tissue specificity of auxin efflux carrier genes. (**A**) Photograph and expression patterns of *McPIN1* and *McPIN3* mRNAs in different organs of bitter gourd. Each lane contained 20 µg of total RNA isolated from different organs. Northern blot analysis was performed with ^32^P-radiolabeled insert of cDNA clone pMAEC43 (for *McPIN1*) and pMAEC93 (for *McPIN3*); (**B**) Hybridization signals were quantified by using LabWorks bioimaging system. The expression level in each treatment was normalized to the 26S and 18S loading control; the maximum level was set to 100%, and all other organ points were normalized to it. Relative expression was determined in triplicate measurements in three independent biological replicates. Error bars represent the standard deviation.

**Figure 5:**
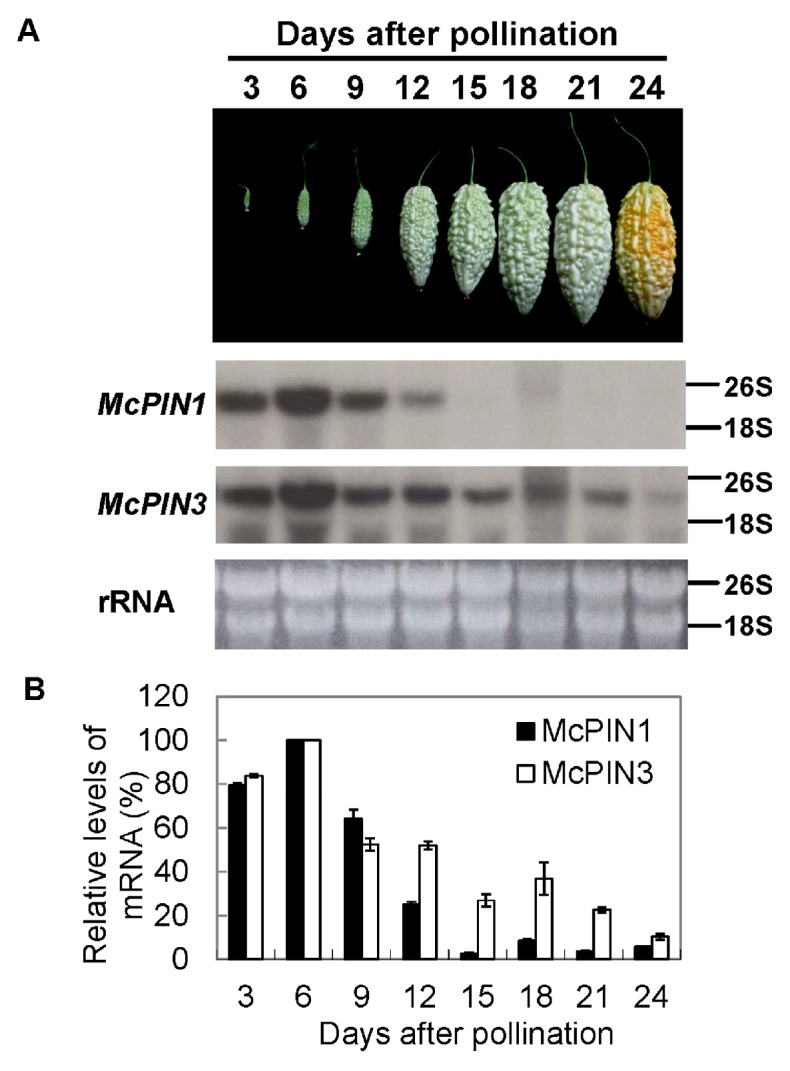
Differential expression of auxin efflux carrier genes during fruit development and ripening of bitter gourd. (**A**) Accumulation of mRNA of auxin efflux carrier genes in bitter gourd during fruit development; (**B**) The expression level in each treatment was normalized to the 26S and 18S loading control; the maximum level was set to 100%, and all other time points were normalized to it. Relative expression was determined in triplicate measurements in three independent biological replicates. Error bars represent the standard deviation.

According to the results of Northern blot analysis, 2.3 kb of mRNA of *McPIN1* was abundant in flowers. There were smaller amounts in roots and stem, while it was not detectable in young or mature leaves. On the contrary, the mRNA of *McPIN3* that was 2.5 kb in length was detected in all tissues of bitter gourd, including roots, stems, leaves and flowers (Figure 4A). Maximum *McPIN1* mRNA accumulated in both staminate and pistillate flower, while maximum *McPIN3* mRNA was found in the staminate flower (Figure 4B). To understand the expression pattern of *McPIN1* and *McPIN3* genes in fruit development and ripening, the mRNA of bitter gourd harvested at different days after pollination was extracted to perform Northern blot analysis. It was classified into four stages: the first stage being 6 days after pollination; the second stage being 12 days after pollination; the third stage being 18 days after pollination; and the last stage being 24 days after pollination. The growth of fruit is halted and the ripening starts at stage 3. Accumulation of mRNA for both *McPIN1* and *McPIN3* genes decreased throughout the fruit development. *McPIN1* mRNA was detectable only in the early stages (stage 1 and stage 2) of fruit development (Figure 5A), whereas the hybridization signal corresponding to the expression of *McPIN3* was still detectable at stage 4 (Figure 5B).

### 2.4. Effects of Exogenous Auxin on Bitter Gourd Auxin Efflux Carrier Gene Expression

**Figure 6:**
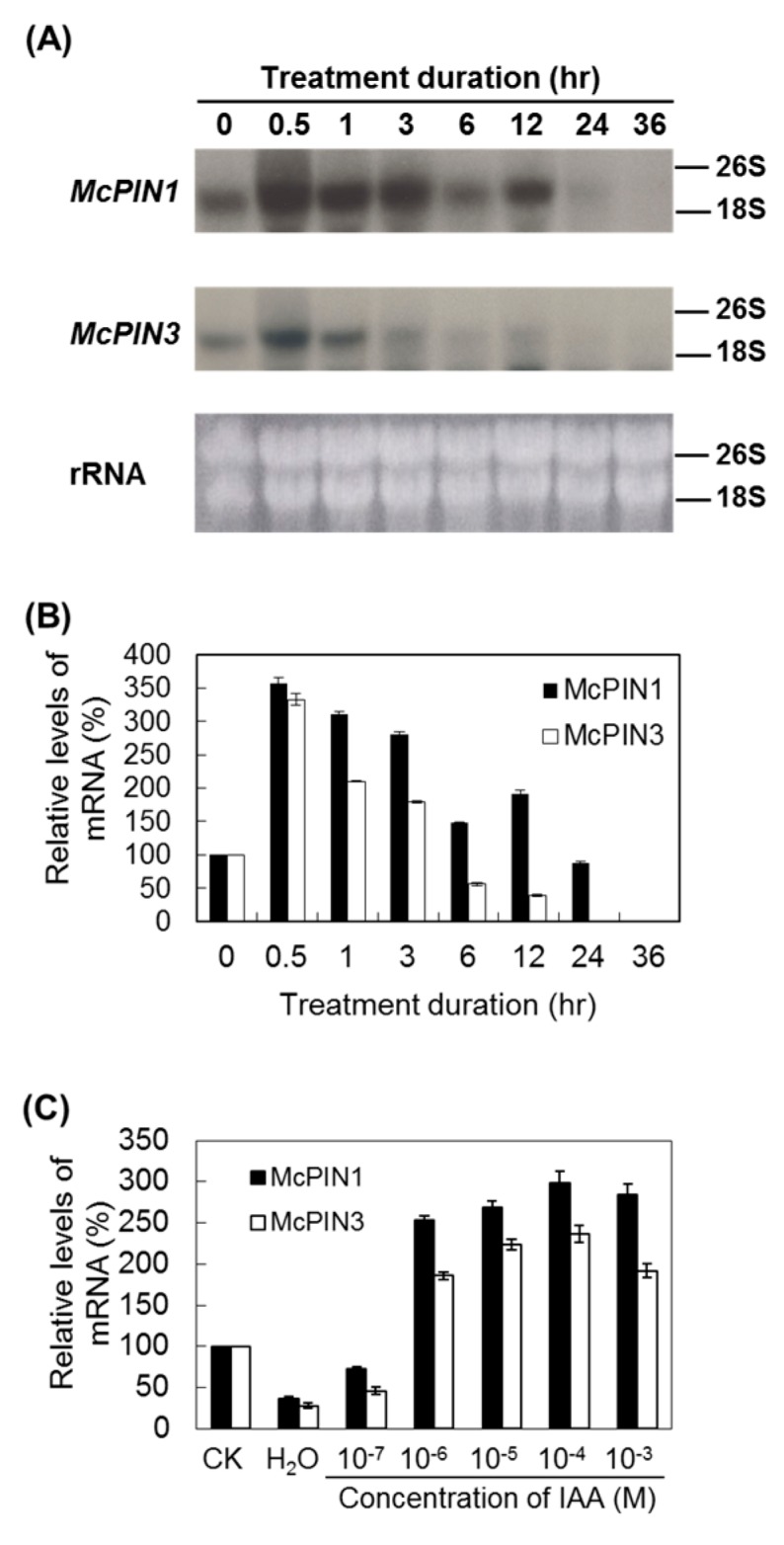
Effects of duration and concentration of indoleacetic acid (IAA) treatment on the accumulation of auxin efflux carrier mRNA. (**A**) Effect of duration of IAA treatment at 10^−5^ M on the accumulation of auxin efflux carrier mRNA. Northern blot analysis was performed with ^32^P-radiolabeled insert of cDNA clone pMAEC43 (for *McPIN1*) and pMAEC93 (for *McPIN3*); (**B**) Hybridization signals were quantified by using LabWorks bioimaging system. The expression level in each treatment was normalized to the 26S and 18S loading control; the level at 0 h was set to 100%, and all other time points were normalized to it; (**C**) Gene expression in response to different concentrations of IAA incubated for 0.5 h in bitter gourd. The expression level in each treatment was normalized to the 26S and 18S loading control; the level at control (CK) was set to 100%, and all other concentration points were normalized to it. Relative expression was determined in triplicate measurements in three independent biological replicates. Error bars represent the standard deviation.

Gene expression of *McPIN1* and *McPIN3* was induced by exogenous IAA. The expression of both genes dramatically decreased with the duration of treatment at different speeds. The accumulation duration of *McPIN1* mRNA was longer than that of *McPIN3*. The effect of enhancement of *McPIN1* gene expression by 10^−5^ M IAA lasted for 12 h, before the mRNA began to degrade 24 h after incubation (Figure 6A,B). Therefore, to determine the optimal concentration for induction of gene expression, the sections of bitter gourd fruit were treated with 10^−3^ to 10^−7^ M of IAA and incubated for 0.5 h. IAA at 10^−6^ to 10^−3^ M levels enhanced the expression of *McPIN1* and *McPIN3* (Figure 6C).

### 2.5. Effects of Exogenous Auxin on Promoter Activity of Auxin Efflux Carrier Genes from Bitter Gourd in Transgenic Tobacco

**Figure 7:**
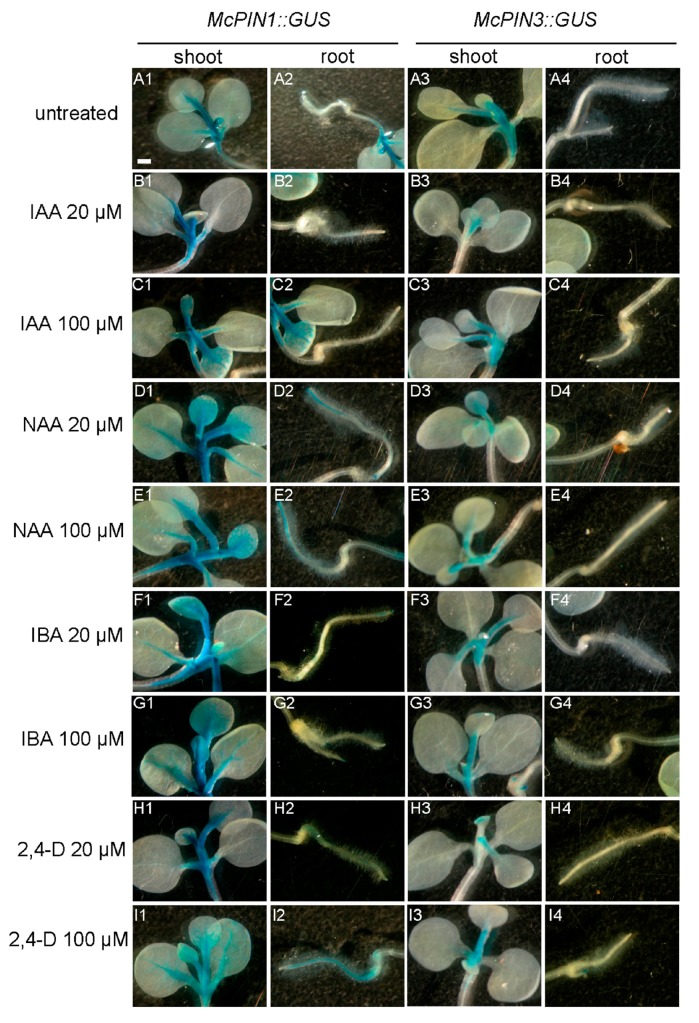
Effect of auxins on the expression in tobacco *McPINpro::GUS* gene promoter transgenic seedlings. Four-week-old seedlings were treated with different kinds of auxins at 20 or 100 µM for 24 h. (**A**) Untreated seedlings as a negative control; (**B**) 20 µM IAA; (**C**) 100 µM IAA; (**D**) 20 µM NAA; (**E**) 100 µM NAA; (**F**) 20 µM IBA; (**G**) 100 µM IBA; (**H**) 20 µM 2,4-D; (**I**) 100 µM 2,4-D. (1) and (2) respectively denote the shoot and root of *McPIN1pro::GUS* transgenic tobacco; (3) and (4) respectively denote the shoot and root of *McPIN3pro::GUS* transgenic tobacco. Scale bars of *McPIN::GUS* as A1, Scale bars: 0.1 cm.

β-Glucuronidase (GUS) activity in both transgenic tobacco seedlings was detected only in leaf veins and were stronger in *McPIN1_pro_::GUS* compared to that in *McPIN3_pro_::GUS* plants. The promoter activity was induced by 1-naphthaleneacetic acid (NAA) and indole-3-butyric acid (IBA) in the mesophyll of newborn leaves. This was enhanced by 20 µM of NAA and 100 µM of 2,4-dichlorophenoxyacetic acid (2,4-d) in the roots of *McPIN1_pro_::GUS* transgenic tobacco. All auxins treated in this study had a slightly suppressive effect on *McPIN3* promoter activity (Figure 7).

## 3. Discussion

This is the first report on the expression patterns of auxin efflux carrier PIN-like genes in monoecious Cucurbitaceae. The gene expression of *CsPIN1* from cucumber was only investigated in roots and it appears to be involved in peg formation [24]. Previous studies did not examine the gene expression of *AtPIN1* or other *AtPIN* genes from *Arabidopsis* in the capsule. Expression patterns of ten tomato *SlPIN* genes were investigated in flower buds and developing fruits [18,19] since the tomato is a hermaphrodite plant similar to *Arabidopsis*. Here, we characterized that *McPIN* genes from bitter gourd belong to a gene family. A similar situation occurred for the genome of other plants, such as eight *AtPIN*s for *Arabidopsis* [9], at least two genes for pea [25], five for potato, six for soybean, eight for wheat, nine for rice [9], ten for *Medicago truncatula* [26], and ten for tomato [18]. All the deduced amino acid sequences of *McPIN1*, *McPIN2* and *McPIN3* exhibited conserved regions in 5′- and 3′-end corresponding to the hydrophobic regions containing transmembrane domains similar to the other auxin efflux carrier proteins, such as *CsPIN1*−*6* (Figure 1). Short *PIN* genes might not express or express traces in developing fruits of bitter gourd so we did not obtain any related cDNA clones. The variability in the length of AtPIN proteins was due to differences in the amino acids in the central region, such as AtPIN5, AtPIN6 and AtPIN8, which lack a central loop domain [9]. Additionally, while compared to the putative amino-terminal signal peptide in AtPIN2, which is presumably cleaved at the end of the second putative transmembrane domain between Ser^60^ and Ser^61^ [10], the potential cleavage site in McPINs is more typical because it is located between the first and the second predicted transmembrane domains (Figure 1). According to the phylogenetic analysis, McPIN1 and McPIN3 share a relatively high sequence similarity with SlPIN1 and SlPIN3/4 [18,19,20], respectively (Figure 2 and Table 2). It indicates that these two auxin efflux carrier proteins in bitter gourd might play similar roles in the fruit development as SlPINs. Furthermore, there are different roles for McPIN1 and McPIN3 in auxin transport involved in the fruit development of bitter gourd. There is no doubt that the closest orthologous relationships occur between the McPINs from bitter gourd and CsPINs from cucumber (Figure 2), which both belong to the monoecious Cucurbitaceae. The numbers of exons and introns in *McPIN1* and *McPIN3* (Figure 3) are the same as in *AtPIN3* and *AtPIN4* [11]. There are five exons in *AtPIN1* [7] and nine exons in *AtPIN2* [27].

The expression level of *McPIN1* is much higher in staminate and pistillate flowers than in roots and stems, while *McPIN1* mRNA can barely be detected in young or old leaves. However, the expression pattern of *McPIN3* (Figure 4) is similar to that of *AtPIN1*, in which mRNA was detected in all *Arabidopsis* organs tested, including cotyledons, flowers, roots, rosette leaves, seedlings, inflorescence axes and siliques [7]. Tomato *SlPIN4* was found to be expressed abundantly in sepal, petal, ovary and young leaves, while it was expressed slightly in mature leaves, roots, and stems. Furthermore, the expression of SlPIN4 increased gradually during the flower development to the flowering stage [19]. The accumulation of *SlPIN1* and *SlPIN9* mRNA was greatest in the ovary, while that of *SlPIN3* and *SlPIN7* was greatest in the petals. The expression levels of the other *SlPINs* were one tenth that of *SlPIN4* [19]. The gene expression pattern of *McPIN3* in different organs of bitter gourd is similar to that of *SlPIN4* in tomatoes.

Both *McPIN1* and *McPIN3* expression were enhanced by exogenous auxin in the fruit of bitter gourd 30 min after treatment (Figure 6). Auxin-mediated cell elongation, one of the fastest hormonal responses known (with a lag period of 15–25 min), is associated with rapid changes in the expression of a select set of early genes [28,29], such as *PS-IAA4/5* and *PS-IAA6* from peas [30]. Therefore, *McPIN1* and *McPIN3* genes qualify as the early auxin-response genes [31]. It suggests that the regulatory functions of McPIN1 and McPIN3 in auxin-induced events are responsible for auxin transportation and cell growth in fruit. On the other hand, only the expression of *McPIN1* promoter was enhanced in the roots of transgenic tobacco (Figure 7). In *Arabidopsis*, the expression pattern of *AtPIN2* in seedlings revealed a root specificity, which correlated well with the root-specific alterations detected in *agr1/eir1/pin2* mutants [10,32]. No *AtPIN2* transcripts were detected in other parts of seedlings or mature plants, although they could be found in hypocotyl and cotyledon tissues of etiolated seedlings at a level 10−fold lower than in roots [32]. Gene expression of *MtPIN2* was limited to nodulated roots, while transcripts of all other expressed genes were detected in both shoots and roots of *Medicago truncatula* [26]. Tomato *SlPINs* were also found to be expressed in roots, although this was at lower levels [18,19,20]. Different group McPIN1 classified by the amino acid sequence-based dendrogram (Figure 2) and the expression patterns (Figure 4 and Figure 7) of AtPIN2 indicated that another McPIN protein is responsible for auxin transport in roots.

The differences of gene expression between *McPIN1* and *McPIN3* are significant during fruit development and ripening. *McPIN1* was expressed in a particular period of early fruit development but *McPIN3* continued to be expressed until the last stage of fruit ripening (Figure 5). The gene expression of *SlPIN4* also decreased during the fruit development in tomatoes [19]. In the fruit of tomatoes, the expression level of *SlPIN4* mRNA was higher in the central part of the fruit (columella, locular tissue) than the outer part of the fruit (exocarp) [18,19]. Moreover, silencing of *SlPIN4* in tomato enhanced precocious fruit development and resulted in parthenocarpic fruits [19]. As we know, auxins stimulate ovule growth and fruit setting is initiated by successful pollination. Specific inhibitors for polar auxin transport, such as 2,3,4-triiodobenzoic acid (TIBA), resulted in accumulation of auxin in the cucumber ovary and production of seedless parthenocarpy fruit without pollination [33]. According to the similar amino acid sequences and expression pattern to *SlPIN4*, McPIN3 could play the same role as SlPIN4 in fruit setting. Since no parthenocarpic fruits have been found in bitter gourd, it would be useful to further investigate the application of exogenous auxin to the unpollinated ovary of bitter gourd or gene silencing that is specific to *McPIN1* or *McPIN3*.

## 4. Materials and Methods

### 4.1. Plant Materials

Bitter gourd (*Momordica charantia* L. cv. Moon Shine) plants were grown in a greenhouse using the King Root Plant Medium No. 3 as substrate. Immediately after harvest, the fruit, stem, leaves, roots and flower tissues were separated, frozen in liquid nitrogen, and stored at −80 °C until use. Sections of sarcocarp tissues (15 mm × 10 mm × 10 mm, 2.5 g) were sampled from bitter gourd 18 days after pollination using a surgical blade under aseptic conditions and were immersed in the solutions of different concentrations (10^−7^, 10^−6^, 10^−5^, 10^−4^ and 10^−3^ M) of IAA for 0.5 h or for different duration of treatment (0, 0.5, 1, 3, 6, 12, 24, 36 h) with 10^−5^ M IAA. All experiments were conducted in the presence of 50 µg mL^−1^ chloramphenicol and 0.1% (*v*/*v*) ethanol. Sections were immersed in the solution containing 50 µg mL^−1^ chloramphenicol and 0.1% (*v*/*v*) ethanol, which served as the control.

### 4.2. RNA Extraction

Total RNA was isolated by guanidium thiocyanate solution (4 M guanidium thiocyanate, 25 mM sodium citrate, pH 7.0, 1% (*v*/*v*) β-mercaptoethanol), which was followed by phenol-chloroform extraction [34]. Poly(A)^+^ RNA was isolated from total RNA by oligo dT cellulose column chromatography [35].

### 4.3. Construction and Screening of cDNA Library and Genomic Library

Poly(A)^+^ RNA was isolated from bitter gourd fruit 16 days after pollination. Double-stranded cDNAs were prepared according to the method described by Kimmel and Berger [36] and inserted into the *Eco*RI and *Xho*I sites of λZAPII using the cDNA Synthesis Kit supplied by Stratagene (La Jolla, CA, USA), which was used to construct the cDNA library. Approximately 1.5 × 10^6^ amplified plaques were screened with *AtPIN1* (517–967 of AF089085) and *AtPIN2* (651–1531 of AF086907) gene fragments synthesized from using *Arabidopsis* as a probe. Hybridization was performed as described in Sambrook et al. [37]. Rescue for the putative clones was accomplished by in vivo excision according to the manufacturer’s protocol (Stratagene) to produce derivatives as pBluescript SK(−) plasmids.

For construction of the genomic library, genomic DNA was extracted from leaves of bitter gourd as described by Jofuku and Goldberg [38]. After partial digestion by *Sau*3AI, DNA fragments of 15–25 kb were collected and inserted into *Bam*HI sites of the λEMBL3 replacement lambda phage vector (Stratagene, La Jolla, CA, USA). Hybridization was performed as described in Sambrook et al. [37] using the pMEC43 and pMAEC93 cDNA as probes.

### 4.4. Nucleotide and Amino Acid Sequence Analysis

Sequencing was performed using an ABI PRISM 377 DNA Sequencer according to the manufacturer’s instructions. Amino acid sequences were aligned using the AlignX program of Vector NTI Suite 10.0 Software (Thermo Fisher Scientific Inc., Waltham, MA, USA). The cleavage site of a potential signal peptide was analyzed by SignalP 3.0 [21]. Polypeptide topology prediction has been performed using the HMMTOP-Server [39].

### 4.5. Southern Blot Analysis

Genomic DNA (20 μg) after single or double-digestion with *Eco*RI, *Bam*HI, *Hin*dIII, and *Sal*I was separated by 0.7% (*w*/*v*) agarose gel electrophoresis, before being transferred onto a nylon filter (Micron Separations Inc., Westborough, MA, USA). The DNA blots were hybridized with the [^32^P]dCTP-labeled probe by random priming [40]. Hybridization was performed overnight at 65 °C in 6× SSPE, 5× Denhardt’s reagent [41], 0.5% (*w*/*v*) SDS, and 250 μg mL^−1^ of salmon sperm DNA. The membranes were washed twice in 2× SSPE, 0.1% (*w*/*v*) SDS at room temperature for 15 min each, before being washed twice in 1× SSPE, 0.1% (*w*/*v*) SDS at 65 °C for 15 min each. The hybridized membrane was exposed at −80 °C using a Kodak XAR-5 film with an intensifying screen for one week.

### 4.6. Northern Blot Analysis

Total RNA (20 μg) was denatured by glyoxylation [42] and electrophoresed through a 1% (*w*/*v*) agarose gel. Transfer of RNA onto a nylon membrane (Micron Separation Inc.) and subsequent hybridization were carried out by conventional methods, as instructed by the manufacturer. The amount of ribosomal RNA was used as a loading control. Relative expression was determined in triplicate measurements in three independent biological replicates.

### 4.7. Promoter Activity Analysis

Wild-type tobacco (*Nicotiana tabacum* L. cv. Wisconsin 38) was transformed to generate *McPIN1_pro_::GUS* and *McPIN3_pro_::GUS* transgenic tobacco. Homozygous transgenic seeds of tobacco (R2) were germinated on the selective media (half-strength Murashige and Skoog (1/2 MS) media [43] containing 100 mL L^−1^ of kanamycin). Transgenic tobacco seedlings were grown in a culture room with cycles of 16-h light and 8-h darkness at 25 ± 2 °C. Four-week-old seedlings were treated with IAA, NAA, IBA, and 2,4-D at 20 or 100 µM for 24 h and analyzed by GUS staining. GUS histochemical staining was performed as described by Jefferson et al. [44].

## 5. Conclusions

Auxin exerts a major influence on transcriptional regulation in plants and other organisms. Auxin efflux carriers are thought to provide an adaptive advantage by allowing auxin transport to tissues where auxin acts to regulate physiological processes. Our approach to understanding the functions of auxin action is to study the regulation of carrier protein genes and then work backward toward the central auxin function. In this study, we investigated the gene expression patterns of the auxin efflux carrier genes during fruit development of bitter gourd. We found that the auxin efflux carrier genes might be involved in the regulation of fruit development. This finding provides novel insights into roles of auxin efflux carriers and a better understanding of fruit growth and the development of juicy melon at the molecular level. Since the function of *SlPIN4* gene has been elucidated previously [19], gene expression during monoecious flower development, protein localization in juicy fruit and gene function of *McPINs* will be further investigated.

## Figures and Tables

**Table 1:** Properties of auxin efflux carrier homologous genes isolated from bitter gourd.

Clone Designation	Size of cDNA (bp)	Accession Number in GenBank	Name of Gene	Number of Amino Acids	Molecular Weight (Da)	Predicted pI Value
pMAEC43	2270	AF247004	*McPIN1*	607	66,385	9.13
pMAEC28	2924	AF246995	*McPIN2*	607	66,300	9.04
pMAEC93	2511	AF247005	*McPIN3*	634	68,821	8.62

**Table 2:** Pairwise comparison of the amino acid sequence identity from auxin efflux carriers from bitter gourd (McPIN1 to 3), and *Arabidopsis* (PIN1, PIN2, PIN3, PIN4, PIN5, PIN6, PIN7, and PIN8) using the AlignX program of Vector NTI Suite 9.0 software. GenBank accession numbers are AF247004 (McPIN1), AF246995 (McPIN2), AF247005 (McPIN3), AF089084 (AtPIN1), AF086907 (AtPIN2), AF087818 (AtPIN3), AF087016 (AtPIN4), AB005242 (AtPIN5), AF087819 (AtPIN6), AF087820 (AtPIN7), and AL391146 (AtPIN8).

%	McPIN1	McPIN2	McPIN3	AtPIN1	AtPIN2	AtPIN3	AtPIN4	AtPIN5	AtPIN6	AtPIN7
McPIN2	99.5									
McPIN3	64.6	64.2								
AtPIN1	73.6	73.1	59.3							
AtPIN2	58.5	58.2	57.7	58.8						
AtPIN3	62.5	62.2	72.5	59.4	55.6					
AtPIN4	64.5	64.2	72.7	60.8	57.7	76.3				
AtPIN5	24.4	23.9	22.2	24.1	23.8	23.0	24.3			
AtPIN6	47.5	47.2	44.3	46.2	46.0	43.9	45.6	25.3		
AtPIN7	62.9	62.6	72.9	59.9	56.5	84.2	76.4	23.6	45.0	
AtPIN8	31.7	31.4	28.6	28.9	27.4	29.6	30.8	30.0	29.9	30.4

## Supplementary Materials

Supplementary materials can be found at www.mdpi.com/1422-0067/18/11/2343/s1.

## Abbreviations

IAA	Indole-3-acetic acid
NAA	1-Naphthaleneacetic acid
IBA	Indole-3-butyric acid
2,4-D	2,4-Dichlorophenoxyacetic acid
