# Supplementary material for: Two Paralogous Genes Encoding Auxin Efflux Carrier Differentially Expressed in Bitter Gourd (Momordica charantia)
Source: Int J Mol Sci. 2017 Nov 6;18(11):2343. doi: 10.3390/ijms18112343 (PMC5713312; doi:10.3390/ijms18112343)
Supplement: Supplementary file 1 [file ijms-18-02343-s001.pdf]

Two Paralogous Genes Encoding Auxin Efflux Carrier Differentially Expressed in Bitter Gourd (*Momordica charantia*)

Yi-Li Li [1], Yun-Shan Lin [1], Pung-Ling Huang [1,2] and Yi-Yin Do [1,*]

[1] Department of Horticulture & Landscape Architecture, National Taiwan University, Taipei 10617, Taiwan; yili2emily@gmail.com (Y.-L.L.); linashango@gmail.com (Y.-S.L.); pungling@ntu.edu.tw (P.-L.H.)
[2] Graduate Institute of Biotechnology, Chinese Culture University, Taipei 11114, Taiwan
* Correspondence: yiyindo@ntu.edu.tw; Tel.: +886-2-3366-4835

```
CCACGCTCTCTCTTGCTTTGAGGACTGCAAGTGCAAGCCTGCTCGCAATTCTCAAAGCTGAATGAATATATTTTTTCTTTCTCTTTTTAT   90
AATTTCCCATTTCCTCTTTTCTTTTTCCACTTAAGATTCACCATTCCCATAAACCAAAACACATGCCCTCTCACTCCCCCAACTCACTTC   180
ATCATCGCCACCACTTCCTCTTCTTCTTCTTCTTCGCTTCCTCTGTTCTGCTTTTCTGAGCTCTCCACAGCCATTTTCTCCATCTGG   270
GTCAGCTCAGAATCCACCAGAAACAAGGAGAAGAAGAAGATGATTAGCCTTTTAGATTTCTACCATGTAATGACCGCAGTGGTTCCTCTT   360
                                               M  I  S  L  L  D  F  Y  H  V  M  T  A  V  V  P  L
TATGTGGCCATGATCTTAGCTTATGGCTCTGTGAAATGGTGGAAAATCTTCACTCCCGACCAATGCTCTGGAATCAACCGTTTCGTTGCT   450
 Y  V  A  M  I  L  A  Y  G  S  V  K  W  W  K  I  F  T  P  D  Q  C  S  G  I  N  R  F  V  A
CTGTACGCGGTCCCTCTTCTCTCTTTTCACTTCATCTCCACCAACAATCCCTATACCATGAACCTCCGCTTTATTGCTGCAGATACTCTC   540
 L  Y  A  V  P  L  L  S  F  H  F  I  S  T  N  N  P  Y  T  M  N  L  R  F  I  A  A  D  T  L
CAAAAACTCATTGGTCTCGCTGTTCTTGCCGTTTGGAGCAACATAAGCAAAAGAGGGTGTTTAGAGTGGACTATTACTCTGTTTTCCCTT   630
 Q  K  L  I  G  L  A  V  L  A  V  W  S  N  I  S  K  R  G  C  L  E  W  T  I  T  L  F  S  L
TCCACTCTGCCAAATACTCTGGTTATGGGGATTCCTCTGCTGAAGGGGATGTATGGGGAGTTTTCTGGGAGTTTGATGGTTCAGATTGTT   720
 S  T  L  P  N  T  L  V  M  G  I  P  L  L  K  G  M  Y  G  E  F  S  G  S  L  M  V  Q  I  V
GTGCTGCAGTGCATTATTTGGTACACATTGATGCTGTTCATGTTTGAATACAGAGGAGCCAGAATGCTGATTTCTGAGCAATTTCCAGAC   810
 V  L  Q  C  I  I  W  Y  T  L  M  L  F  M  F  E  Y  R  G  A  R  M  L  I  S  E  Q  F  P  D
ACTGCTGGCTCCATAGTCTCAATCCATGTCGATTCTGACATTATGTCGCTGGATGGAAGGCAAGTTTTGGAAACAGAGGCTGAGATTAAA   900
 T  A  G  S  I  V  S  I  H  V  D  S  D  I  M  S  L  D  G  R  Q  V  L  E  T  E  A  E  I  K
GAAGATGGGAAGCTCCACGTGACTGTGAGGAAATCAAACGCCTCAAGATCGGACATCTTCTCCAGAAGATCTGTGGGGTTGTCTTCTACA   990
 E  D  G  K  L  H  V  T  V  R  K  S  N  A  S  R  S  D  I  F  S  R  R  S  V  G  L  S  S  T
ACCCCACGGCCTTCCAATCTGACCAATGCCGAGATTTACTCTCTGCAATCTTCTCCAAACCCCACTCCCAGAGGATCAAGTTTTAACCAC   1080
 T  P  R  P  S  N  L  T  N  A  E  I  Y  S  L  Q  S  S  P  N  P  T  P  R  G  S  S  F  N  H
ACGGATTTCTACTCCATGATGGCCGCCGGTGGAGGAATTCCAATTTCGGCTCATCGGATGTTTACGGGCTCTCCGCGTCGAGAGGGCCG   1170
 T  D  F  Y  S  M  M  A  A  G  G  R  N  S  N  F  G  S  S  D  V  Y  G  L  S  A  S  R  G  P
ACTCCGAGGCCGTCAAATTATGAGGAAGATGGTGGGGGAAAGCCGAGGTTTCACTACAATGCTGCGGCGGGAGGCAATGCAGGGCACTAC   1260
 T  P  R  P  S  N  Y  E  E  D  G  G  G  K  P  R  F  H  Y  N  A  A  A  G  G  N  A  G  H  Y
CCTGTTCCGAACCCAGGAATGTTTTCGCCAACGGGGTCGAAAAATGCACAACCCAATGCTAAGAAGTCTGCCAATGGCAATGGGGTTGTG   1350
 P  V  P  N  P  G  M  F  S  P  T  G  S  K  N  A  Q  P  N  A  K  K  S  A  N  G  N  G  V  V
GCTCAAAAAACAGAGGAAGGAAACAGAGATCTCCATATGTTTGTTTGGAGTTCAAGTGCTTCCCCTGTTTCAGATGTTTTTGGGGGAAAC   1440
 A  Q  K  T  E  E  G  N  R  D  L  H  M  F  V  W  S  S  S  A  S  P  V  S  D  V  F  G  G  N
CATGAATTTGGGGGAAACCATGATCAGAAGGATGTGAGGTTGGCTGTCTCCCCCGGAAAAGTTGAGGGTCGTAGAGAAAACCAAGAGGAG   1530
 H  E  F  G  G  N  H  D  Q  K  D  V  R  L  A  V  S  P  G  K  V  E  G  R  R  E  N  Q  E  E
TATGCAGAGAGGGAAGATTTCAGCTTCGGAAACAGAGAGATTCTGAATAATAACAATGGAGGAGCAGAGAAAGTTGGAGATATTAAACCC   1620
 Y  A  E  R  E  D  F  S  F  G  N  R  E  I  L  N  N  N  N  G  G  A  E  K  V  G  D  I  K  P
AAAACCATGCCACCCACCAGTGTAATGACAAGGCTCATTCTGATCATGGTTTGGAGAAAACTCATCAGAAACCCCAACACTTATTCCAGC   1710
 K  T  M  P  P  T  S  V  M  T  R  L  I  L  I  M  V  W  R  K  L  I  R  N  P  N  T  Y  S  S
CTAATTGGCCTCACTTGGTCCTTAGTCTCATTCAGGTGGAATGTTGAAATGCCAGCCATTGTCGCAAAGTCCATTTCTATACTGTCTGAT   1800
 L  I  G  L  T  W  S  L  V  S  F  R  W  N  V  E  M  P  A  I  V  A  K  S  I  S  I  L  S  D
GCAGGCCTCGGCATGGCCATGTTCAGTCTTGGTTTGTTCATGGCTTTGCAACCAAGGATCATAGCATGTGGGAATACAATAGCAGCTTTT   1890
 A  G  L  G  M  A  M  F  S  L  G  L  F  M  A  L  Q  P  R  I  I  A  C  G  N  T  I  A  A  F
TCCATGGCTGTGAGATTCCTTACAGGTCCAGCTGTCATGGCTGTTGCTTCCATTGCTGTTGGCCTTAGAGGAGTTCTCTTACGTGTTGCC   1980
 S  M  A  V  R  F  L  T  G  P  A  V  M  A  V  A  S  I  A  V  G  L  R  G  V  L  L  R  V  A
ATTGTCCAGgtcacccattcgctccctctctgacactccctcgttcctttttctctgcatatactcaaaaaatcctctctttaatatata   2070
 I  V  Q
catttataatacacactgtaaagcagagtttatccaaatgggtaatggaaaaaaaatcttctttcttcttgaaggtaaataataagcaaa   2160
acaagaacagaggggttaaatgaaaacttaccctattaatttcatactaatctcattcattgtttgccatgatcagtattgaatctgaaa   2250
tagaaatagtatgagttgtgaggtaatataggtgaggagaggacctgtttggtgataagttaggaaacaaacatctgtggctctgtttaa   2340
catgtttttttgccccatgtgtttggattttccaaacacaaccacatctacaaaaaattctttgtttttttgtttggaacagGCAGCTCTC   2430
                                                                                   A  A  1
CCACAAGGAATTGTCCCCTTTGTCTTTGCCAAAGAGTACAACGTACACCCTGATATTCTCAGCACAGGAGTTATTTTTGGGATGTTGATT   2520
 P  Q  G  I  V  P  F  V  F  A  K  E  Y  N  V  H  P  D  I  L  S  T  G  V  I  F  G  M  L  I
GCCTTGCCCATTACGCTTGTGTACTACATTTTGCTGGGGATCTGAAGGTCAGCCATGGCTGCGGCTGCGGCCGCCATGAGAGGGCATTTC   2610
 A  L  P  I  T  L  V  Y  Y  I  L  L  G  I  *
TAAAGCTTCAATTGGCTTTCGGGAAAGAAGATTTTAGAGGGGGGAGAAGATGGAGGAAGTCAAATGGGGAAGGTGAAAGAGGAGAGGATT   2700
GAGGAATTTGGGATGAATGTGTGAATCTTCTTTCCAAGTAAAAGAAAAAAGAAAAAGGGGCTGTAAAATCATGATGTTTGGTAGTCAATT   2790
ATATAAGAAAAAAAAGTGTTTTTGATTTTAAAAAAAAAAAAAAAAAAAAA                                           2840
```

Figure S1.
